# Validation of the Short Parallel and Extra-Short Form of the Heidelberg Figural Matrices Test (HeiQ)

**DOI:** 10.3390/jintelligence12100100

**Published:** 2024-10-14

**Authors:** Vanessa S. Pallentin, Daniel Danner, Sven Lesche, Jan Rummel

**Affiliations:** 1 Department of Psychology, Heidelberg University, Hauptstr. 47-51, 69117 Heidelberg, Germany; sven.lesche@psychologie.uni-heidelberg.de (S.L.); jan.rummel@psychologie.uni-heidelberg.de (J.R.); 2 Psychology Section, University of Applied Labour Studies, Seckenheimer Landstr. 16, 68163 Mannheim, Germany; daniel.danner@hdba.de

**Keywords:** figural matrices test, intelligence, reasoning, item response theory, parallel test forms

## Abstract

Figural matrices tests are frequently used to measure fluid intelligence. The HeiQ—an operation-oriented figural matrices test—was developed to tackle limitations of previous matrices tests, mainly the possibility of excluding distractors based on superficial features instead of actively solving the items. However, allowing for a total administration time of 60 min for the assessment of one construct is not feasible in many study designs. Thus, the goal of this study was to develop three short forms of the existing HeiQ. Two parallel 20-item short forms (the HeiQ-S A and HeiQ-S B) that are comparable in content as well as on a psychometric basis and a 6-item short form (the HeiQ-XS) were generated. All tests showed good internal consistency (Cronbach’s Alpha ranging from α = 0.82 to α = 0.86) and good criterion-related validity (correlations with high school grade (Abitur) ranging from *r* = −0.34 to *r* = −0.38); construct validity (correlations with the global intelligence scores of the Intelligence Structure Test 2000R were between r = 0.58 and r = 0.71). Further, all test versions showed to be Rasch-scalable, implying a uniform underlying ability. Thus, we conclude that all three newly developed short versions are valid tools for assessing fluid intelligence.

## 1. Introduction

Fluid intelligence, also called fluid reasoning, is one of the best predictors of educational success and job performance (Deary et al. 2007; Schmidt and Hunter 2004; Schneider et al. 2020). It can be defined as the ability to solve novel problems and detect underlying rules and patterns without relying on previously acquired scripts or knowledge (Carroll 1993; Cattell 1963; McGrew 2009; Schneider and McGrew 2012). Thus, fluid reasoning tests are a sought-after task not only in research but also for admission and education purposes (Schneider et al. 2020). A popular way to assess fluid reasoning is via a figural matrices test. Figural matrices tests can be seen as the best single indicators of general intelligence (Arendasy and Sommer 2013) and are also an integral part of comprehensive cognitive ability test batteries like the Wechsler Adult Intelligence Scale (WAIS-IV) (Wechsler 2012) or the Intelligence-Structure-Test (I-S-T) 2000R (Liepmann et al. 2001).

**Figure 1:**
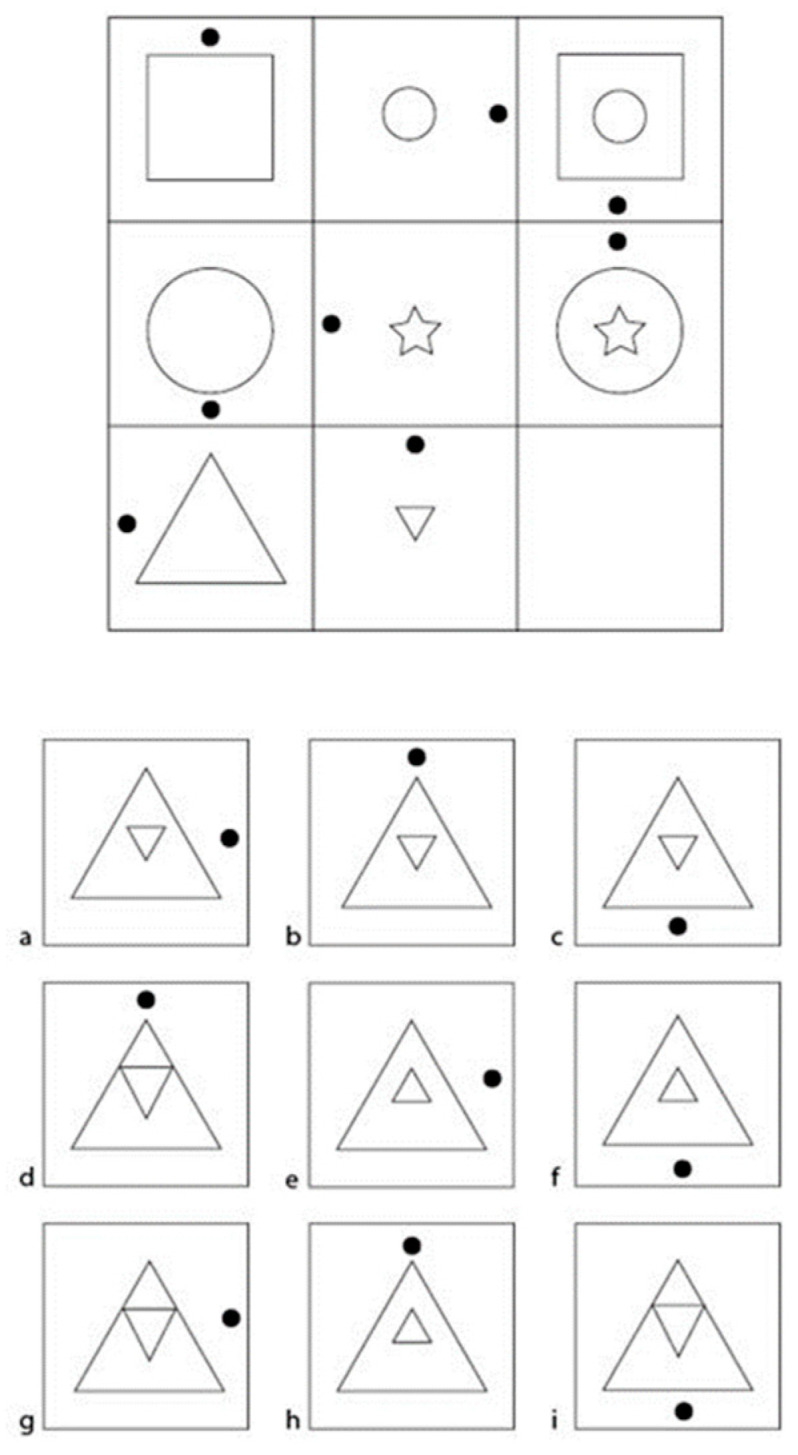
Illustration of an example item from the HeiQ. Subfigure (**a**) correctly follows the two operations, addition and seriation and is the correct response option. All other response options (subfigures **b**–**i**) are distractors.

In a figural matrix task, participants are usually presented with a 3 × 3 matrix—the item stem—containing geometrical forms, elements, and patterns (i.e., arrows, lines, shapes). One cell, typically the bottom right one, is left empty (Heydasch 2014; Raven 1976). The figures in the matrix are arranged in accordance with certain underlying rules or operations that can be applied row-wise, column-wise, or both, and it is the participant’s task to detect these rules and apply them to the empty cell (Formann 1973). Participants are usually given an array of response options and have to identify the response option that correctly completes the matrix following the rules they detected. One response option is correct (the attractor), while the other response options, although plausible, are incorrect representations (the distractors). An example is given in Figure 1. Here, two operations (addition and seriation) are present. For the addition, the figures of the first and second cells are added to form the one in the third cell. For the seriation, figures are systematically altered from one cell to the next—in this case, a clockwise rotation of 90 degrees of the black dot (Hornke and Habon 1986; Hornke et al. 2000).

One shortcoming of many figural matrices tests is the possibility of using response elimination strategies to exclude distractors that are deemed implausible based on superficial features (Arendasy and Sommer 2013; Gonthier and Roulin 2020; Pallentin et al. 2023) instead of mentally constructing the correct response (called constructive matching). Response elimination describes that participants inspect the given response options and try to eliminate those that seem unlikely, for instance, distractors that are visually dissimilar to other response options (Arendasy and Sommer 2013; Becker et al. 2016; Bethell-Fox et al. 1984). For example, if only one of eight distractors features an arrow pointing upward, while the other distractors show an arrow pointing left or right, that distractor would be deemed implausible and, thus, be easily eliminated by participants. Response elimination can go as far as participants identifying the correct response without identifying any of the underlying operations correctly.

A specific strategy of response elimination is called counting (Mittring and Rost 2008). Hereby, figural elements of all distractors are counted based on the number of appearances, and the response options that feature the highest number of “recurring” elements are chosen. For example, for a matrix item, eight response options are given that feature two distinct shapes that represent two operations (circles and squares). Four response options contain three squares, while two response options contain one square, and one response option contains either zero or two squares. Thus, it would be deemed most likely that one of the response options with three squares is correct, as they are present the most. For the circles, a similar pattern emerges, where three response options show two circles, and all other options are fewer in numbers. Thus, it would be deemed most likely that the correct response should also contain two circles. In the last step, the two elements are added, leaving the response option with three squares and two circles most likely. In counting, this response option will be chosen.

Mittring and Rost (2008) have shown that, by applying counting, the correct response option could be identified without having seen the item stem in 50% of the items of the Raven Advanced Progressive Matrix (RAPM) (Raven 1976). Moreover, response elimination, in general, has been shown to coincide with less time spent on the item stem and less conceptual analysis and goal monitoring, both integral elements of the solution process (Becker et al. 2016; Carpenter et al. 1990). As a result, response elimination strategies can be a serious threat to validity, and preventing response elimination has been shown to increase construct and, specifically, convergent validity (Arendasy and Sommer 2013; Becker et al. 2016). To counter counting and the use of response elimination in general, the HeiQ—a 48-item figural matrices test—has been introduced, where all figural elements appear equally often over the given response options of an item, making it impossible for participants to eliminate any distractor without solving at least one of the underlying operations. Furthermore, the distractor design of the HeiQ allows for a more detailed analysis of underlying processes when an incorrect response option is chosen by participants. As there is exactly one distractor representing every possible combination of correctly and incorrectly applied operations, it can be inferred which operations were and were not correctly solved (Pallentin et al. 2023).

One drawback of the HeiQ, however, is the total administration time of 60 min, rendering it unsuitable for studies in which a quick assessment of fluid intelligence is required (Bors and Stokes 1998; Sefcek et al. 2016). A variety of short forms of existing figural matrices tasks have been developed in the past, such as an 18-item short form of the RAPM (Sefcek et al. 2016), two 12-item short forms of the RAPM (Arthur and Day 1994; Bors and Stokes 1998), or a 6-item short form of the Hagen Matrices Test (HMT) (Heydasch 2014; Heydasch et al. 2013), emphasizing the need for more time-efficient measures.

Besides administration time issues, administration repetition issues may occur in many study contexts in which matrices tests are typically applied. When the same test items are used repeatedly in a longitudinal study, observed improvements from one administration to the next one can be either due to a mere retest effect or due to an actual improvement in fluid intelligence (Jendryczko et al. 2019; Lievens et al. 2007). To reduce the risk of retest effects, parallel test versions (i.e., test versions that measure the construct with comparable precision) are required (Jendryczko et al. 2020). Thus, to cater to these issues while keeping the benefits of the HeiQ (Pallentin et al. 2023), we decided to create two shorter parallel versions (HeiQ-S A and HeiQ-S B) with 20 items each and an extra short version (HeiQ-XS) of 6 items.

## 2. Materials and Methods

### 2.1. Participants

For the present study, we used the data from the previously published HeiQ validation study (Pallentin et al. 2023). These are pooled data from students, as well as general German population subsamples. Participants were recruited through various channels, including lectures, university participant pools, and newspaper advertisements. Data were collected both online via the online survey platform SoSci Survey (Leiner 2019) and in a laboratory setting between March 2020 and December 2021. All participants completed the HeiQ and additional tasks specific to their subsample. An overview of the different subsamples, their demographics, and the assessed constructs of interest is given in Table 1.

In total, *N* = 733 participants completed the HeiQ. To ensure data quality, data were cleaned on the following grounds: participants stating they did not follow the instructions (*N* = 13); not solving a very easy-to-catch item (*N* = 6); taking more than 4 h to answer the test items (*N* = 13); having overly fast responses (less than 5 s per item can be seen as a cut-off, where participants can be expected to no longer examine, understand, and answer an item (Wise 2017)) on 50% of all test items (*N* = 18).

The final sample consists of *N* = 683 participants. At the time the data were collected, participants’ mean age was *M* = 25.88 (SD = 9.04); 66.2% were female; 32.7% were male, and 0.3% identified as non-binary or preferred not to say. The majority of participants (84.3%) were university students. A subsample of *N* = 216 participants was part of a longitudinal study that also completed the Berlin Intelligence Structure Test (BIS-S) (Jäger et al. 1997). Out of this subsample, *N* = 205 completed the HeiQ twice. Another subsample of *N* = 76 participants completed the Intelligence Structure Test 2000R (I-S-T 2000R) (Liepmann et al. 2001). High school grade point average (GPA) was used as an additional measure and academic achievement indicator. Here, all participants who reported a German high school qualification (Abitur) were included to ensure comparability of grades.

### 2.2. Development of Two Parallel Versions of the HeiQ (HeiQ-S A and HeiQ-S B)

Previous matrices test short forms have mostly been developed based on empirical–statistical considerations. For example, items that have shown the highest item–total correlation (Bors and Stokes 1998) or the highest correlation with other intelligence tasks (Heydasch et al. 2013) have been selected. For the HeiQ short versions, we aimed to (a) keep all benefits of the original version of the HeiQ—namely, to prevent counting and make sure that, as in the original version of the HeiQ, each operation included the same number of times—and (b) to generate two versions that were not only parallel on a psychometric level but were also content-wise, hence featuring the same operations. Thus, it was our goal to develop a method of item selection that took into account conceptual and psychometric considerations and—in an iterative process—found the best pair of test versions without sacrificing any test properties.

**Figure 2:**
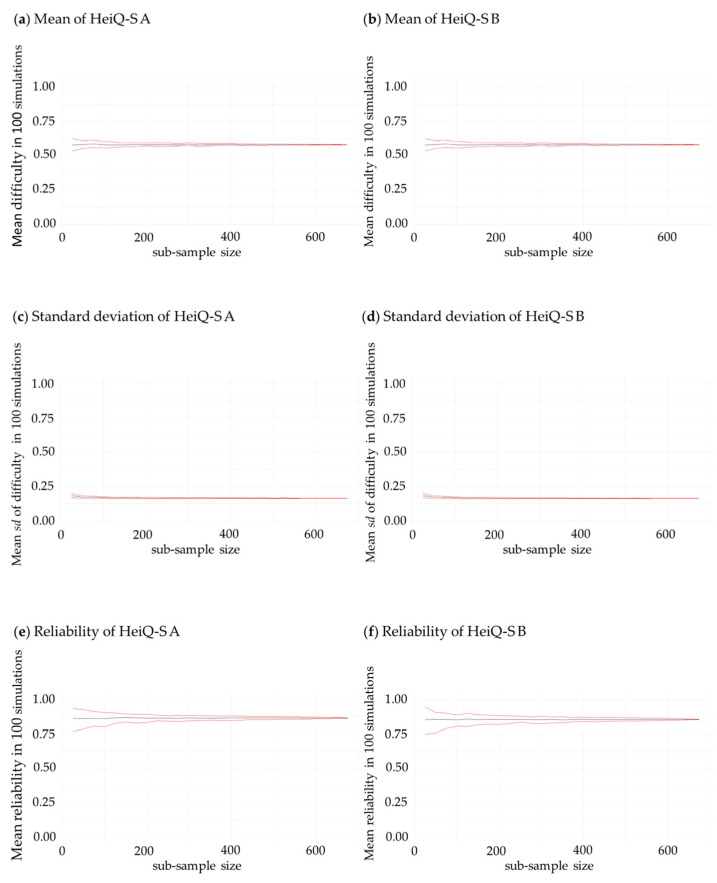
Black lines represent the mean value over 100 resampling iterations. Red lines represent +/− one standard deviation for the mean difficulty and the standard deviation of the difficulty (Graphs (**a**–**d**)) and the 2.5% and 97.5% quantiles for the reliability (Graphs (**e**,**f**)).

In the original version of the HeiQ, there are 24 items which are constructed based on two operations (e.g., addition and seriation in Figure 2) and 24 items which are constructed based on three operations. Among the 24 two-operation items, there are 12 unique combinations of operations. Hence, there are always two items featuring the exact same combination of operations. Among the 24 three-operation items, there are 8 unique combinations of operations, that is, three items featuring the exact same combination of operations. In the first step, we grouped all items that followed the same operations. For the two-operation items, this resulted in 12 item pairs; for the three-operation items, this resulted in 8 item triplets. In order to create two parallel test versions with comparable operation combinations, we intended to end up with 12 two-operation items and 8 three-operation items (i.e., 8 three-operation items were not used for this purpose).

In addition to operation parallelization, we intended to achieve psychometric parallelization, which implies, according to Classical Test Theory, that test variances and reliabilities shall be equal across parallel versions of a test (Gibson and Weiner 1998; Lord et al. 1968). We searched for optimal test versions by exhaustively testing all possible parallel test versions of the two-operation and the three-operation items and computing their respective means, variances, and reliabilities. Reliability was estimated using Spearman–Brown corrected split-half correlations of an odd–even split. For the two-operation items, there are 2^12 = 4096 possibilities to split up the pairs into two-item sets. To illustrate, when item pairs *A1* and *A2* and *B1* and *B2* are split up, the resulting Set 1 could hold four combinations (*A1B1*; *A1B2*; *A2B1*; *A2B2*). As Set 2 always contains all items that are not used in Set 1, there are 2048 possible pairs of sets and, thus, 2048 possible ways to generate two parallel test versions. We computed test means for every version and selected those 150 pairs of parallel test versions that showed the smallest difference in test mean. We decided to choose 150 pairs so that there was still enough variance within the next selection step. We then calculated variances for all versions and kept those 50 pairs of test versions with the smallest difference in test variance. We then selected the best 5 pairs of test versions with the smallest difference in reliability.

The same procedure was applied to the three-operation items, only that all possible combinations of using two out of three items per triplet were assigned to Set 1 or Set 2, and the respective third item was then dropped. After identifying the best 5 pairs of test versions with the smallest difference in mean, variance, and reliability for the three-operation items, we combined them with the top 5 pairs of parallel test versions of the two-operation items to generate pairs of parallel test versions with 20 items each. Of the resulting 50 possibilities, we re-estimated the reliability over the whole test with 20 items and ensured that the difference in reliability between the pairs of parallel test forms was lower than 0.01. This cut-off ensured similar reliabilities while also allowing to take into account other psychometric properties of the test. We then selected the 10 full parallel test versions with the highest average reliability for the two test versions. The R-Syntax for the item selection procedure can be found on the Open Science Framework (OSF; https://osf.io/cxzmv/?view_only=5d9790bd2c65424abbd8fbba2ff68372; uploaded on 5 June 2024).

We aimed to generate shorter test versions that are Rasch-scalable, as this was also a feature of the HeiQ long version. In a Rasch model (Rasch 1960), all items load equally strongly on the latent construct, suggesting that the construct is uni-dimensional. We fit a Rasch model to each of the 10 possible parallel-test versions and selected the two with the highest mean model fits and, at the same time, the smallest difference in model fit. In doing so, we believe that we selected the optimal pair of parallel test versions with regard to psychometric and operation parallelization. In accordance with the average per-item response time of 1:15 min for the HeiQ, as well as similar response times for the HeiQ-S A and HeiQ-S B, we suggest a time limit of 25 min for the HeiQ-S (A or B). This time limit is suggested for the test as a whole, allowing participants to spend as much or as little time on any single item as desired.

### 2.3. Construction of an Extra Short Version of the HeiQ (HeiQ-XS)

Although the HeiQ-S is considerably shorter than the original form, some testing situations may require an even shorter assessment of fluid intelligence. We, thus, generated another 6-item short version of the HeiQ (HeiQ-XS). As there are 20 unique combinations of operations in the HeiQ (12 for the two-operation items and 8 for the three-operation items), it is not possible to keep a balanced operation design with less than 20 items. Thus, the 6-item short version no longer allows for a detailed analysis of performance on the operation level, as is the case for the long version (see Pallentin et al. 2023) and the 20-item versions. However, the main benefit of the HeiQ—that no distractors can be excluded based on superficial features—remains true for the very short version.

Similar to previous constructions of short versions, we chose those items with the highest item–total correlation (Bors and Stokes 1998). Here, items were ranked according to their part-whole corrected item–total correlation, and the best six items were selected. The resulting 6 items showed item–total correlations between *r* = 0.57 and *r* = 0.66, with a mean item–total correlation of *r* = 0.61. These values exceed common cut-off points for acceptable item–total correlations such as *r* = 0.30, as suggested by Ferketich (1991) or Cristobal et al. (2007), and can be interpreted as good indicators of item discrimination (Moosbrugger and Kelava 2012). As the HeiQ-XS is more difficult, we suggest a 10-min time limit, allowing for more average time per item.

An overview of the characteristics of the full version of the HeiQ, the two parallel test forms HeiQ-S A and HeiQ-S B, and the HeiQ-XS is given in Table 2.

### 2.4. Berlin Intelligence Structure Test Short Form (BIS-S)

The short form of the Berlin Intelligence Structure Test (BIS-S) (Beauducel and Kersting 2002; Jäger et al. 1997) measures general cognitive ability and was used to evaluate the construct-related validity of the HeiQ-S (A and B) and HeiQ-XS. The BIS-S consists of 15 tasks designed to assess various mental processes, including reasoning, creativity, memory, and speed across verbal, numerical, and figural domains. Administrating the BIS-S typically takes between 45 to 60 min. In our sample, *N* = 216 participants completed the BIS-S in addition to the HeiQ.

### 2.5. Intelligence Structure Test 2000R (I-S-T 2000R)

The Intelligence Structure Test 2000R (I-S-T 2000R) (Liepmann et al. 2001) was employed as an additional measure of general cognitive ability to evaluate the construct-related validity. The I-S-T 2000R tests three primary domains of cognitive abilities: reasoning; knowledge; and memory. Each of these domains can be further divided into three subcategories: numeric; verbal; and figural. Moreover, from the reasoning and knowledge components of the test, fluid intelligence (*gf*) and crystal intelligence (*gc*) are derived. In our sample, *N* = 76 participants completed the BIS-S in addition to the HeiQ.

## 3. Results

### 3.1. Test Performance

For the two parallel test versions, HeiQ-S A and HeiQ-S B participants solved an average of *M* = 11.74 items (*SD* = 4.83; *Range* = 1–20) and *M* = 11.52 (*SD* = 4.64; *Range* = 1–20), respectively. The effect size of the mean difference in the two versions is *d* = 0.09, which can be seen as a small effect (Cohen 1988). Item difficulties range from 0.28 to 0.93, with an average of 0.60 in both versions. For the HeiQ-XS, participants solved an average of *M* = 2.54 (*SD* = 2.14; *Range* = 0–6) items. An overview of test performance for the original version of the HeiQ, the two parallel short versions, and the 6-item extra short version is provided in Table 3.

### 3.2. Reliability

As a measure of internal consistency, Cronbach’s Alpha was calculated for all test versions. Due to missing data, listwise deletion led to an overestimation of Alpha. As a result, pairwise deletion was imposed. Cronbach’s Alpha was α = 0.86 for the HeiQ-S A, α = 0.85 for the HeiQ-S B, and α = 0.82 for the HeiQ-XS. The split-half correlations, computed as an odd–even split, were *r* = 0.76 and *r* = 0.75 for the HeiQ-S A and HeiQ-S B, respectively (Spearman–Brown corrected). As a minimal difference in reliability estimates was one of the criteria for item selection for the parallel test forms, both tests showed similar reliability. For the HeiQ-XS, the split–half correlation was *r* = 0.70. A subsample of *N* = 205 took the HeiQ twice, with an average test–retest interval of *M* = 87 days (*SD* = 19). The retest correlation was *r* = 0.81 for the HeiQ-S A, *r* = 0.80 for the HeiQ-S B, and *r* = 0.73 for the HeiQ-XS. A detailed overview of all reliability estimates for the three test versions, as well as the original test, is given in Table 4. All tests show good to excellent reliability.

### 3.3. Validity

The construct-related validity of the parallel test versions and the six-item short version was assessed by correlating test results with a variety of other indicators of general cognitive ability, such as the Berlin Intelligence Tests (BIS-S) and the Intelligence Structure Test (I-S-T 2000R). All measures were scored following the scoring procedure in the respective test manuals. The correlations of the HeiQ-S A and B versions were *r* = 0.53 and *r* = 0.57, respectively, with the BIS-S and *r* = 0.69 and *r* = 0.71 with the I-S-T 2000R. The correlations of the HeiQ-XS was *r* = 0.56 with the BIS-S and *r* = 0.58 with the I-S-T 2000R. Thus, all versions show satisfactory correlations with other indicators of cognitive ability, considering the reduced number of items (see Table 5).

We also correlated the 20-item HeiQ-S and 6-item HeiQ-XS with the 48-item HeiQ scale scores to evaluate to what extent we lose information by shortening the scales. The correlation between the HeiQ and the HeiQ-S (and B) was *r* = 0.96. The correlation between the 48-item HeiQ and the 6-item HeiQ-XS was *r* = 0.87 (r* = 1.00 when corrected for attenuation based on Cronbach’s Alphas).

Finally, we estimated the criterion-related validity based on the association with high school grade point average (GPA). To ensure comparability of GPA, only German high school qualification (Abitur) was used. A total of *N* = 472 participants reported a valid GPA. In Germany, a lower number equals a better grade, and a negative correlation between the HeiQ and GPA is expected. Correlation coefficients were similar for all versions of the HeiQ and were between *r* = −0.34 and *r* = −0.39. Similar to Heydasch (2014), correlation coefficients were also calculated for participants under the age of 24 to investigate a more homogenous sample and control for any cohort effects. Here, correlations were also in a similar range, ranging from *r* = −0.47 to *r* = −0.49.

### 3.4. Measurement Models and Robustness Check

A Rasch (1PL) model (Rasch 1960) was applied to test for the uni-dimensionality of the underlying construct. For HeiQ-S A and HeiQ-S B, the item selection included Rasch scalability, as explained in the test generation procedure. We further applied a Rasch model to the HeiQ-XS. Measurement models were estimated using the means and variance-adjusted weighted least square estimator (WLSMV) implemented in Mplus 8.6 (Muthén and Muthén 2017). An overview of the original version of the HeiQ, the two parallel forms, and the HeiQ-XS is given in Table 6. Results show that all tests show acceptable-to-good model fit according to conventional cut-off criteria, supporting the uni-dimensionality of the test (Hu and Bentler 1999; Wheaton et al. 1977; Yu 2002).

We ran robustness checks to ensure that our results regarding the psychometric similarity of the parallel test halves were valid outside of our initial sample. To achieve this, we repeatedly run all analyses with randomly drawn subsamples out of the total sample. In particular, we generate subsamples by drawing *n* participants out of the calibration sample without replacement. In order to provide a complete picture of the impact the number of participants *N* of the new subsample has, we conducted this analysis with *N* ranging from *n* = 25 to *n* = 675, increasing in steps of 25. We then calculate the means, variances, and reliabilities of our proposed parallel test versions in this new sample. This process was repeated 100 times for each sample size. This resulted in a total number of 27 subsamples. The means, the 2.5% and 97.5% quantiles of the means, variances, and reliabilities of the HeiQ-S A and HeiQ-S B are presented in Figure 2. The mean average score, mean standard deviation, and mean reliabilities proofed to be stable, even when only *n* = 50 participants are drawn. Thus, both versions, the HeiQ-S A and the HeiQ-S B, can be considered robust regarding their psychometric properties.

## 4. Discussion

Three shorter test versions of the HeiQ, two parallel test versions, HeiQ-S A and HeiQ-S B, and an extra-short form, the HeiQ-XS, were generated. The HeiQ-S A and HeiQ-S B consist of 20 items each. The HeiQ-XS offers an even more efficient assessment of cognitive ability with six items. With the generation of the parallel test forms, we followed a new approach, not only taking into account psychometric parallelization but also operation parallelization. Both short test forms feature items with the same combination of underlying operations. As a result, every item in the short test version A has a twin item in the short test version B with identical underlying operations but different visual appearances. Thus, it can be expected that the cognitive demands imposed by the two short test versions are comparable. In particular in repeated measurement designs, this is a big advantage as participants can be tested with the parallel test versions at different measurement occasions thereby reducing from the risk of retest effects such as remembering specific items or response options from the first to the second application.

Additionally, we intended to ensure psychometric comparability by splitting up content-aligned item pairs in a way that test means, variances, and reliabilities are as similar as possible. With a small effect size of *d* = 0.09, test means, even though not equal, can be seen as comparable. On top of the psychometric parameters already taken into account in item selection, a high Cronbach’s Alpha and test–retest correlation coefficient further support the reliability of both versions. Even though tests with fewer items usually show a drop in conventional reliability estimates, parameters of the HeiQ-S A and HeiQ-S B are still comparable to the original 48-item version of the HeiQ, further speaking to the success of our item selection approach.

The HeiQ-XS was developed to offer an even more efficient assessment of cognitive ability. With only six items and a suggested application time of 10 min, it is similar or even shorter in time to other short versions of the figural matrices test. The HeiQ-XS also shows relatively good reliability estimates. Nevertheless, it needs to be stated that reliability estimates are smaller than those of the other test versions. Thus, especially when participants are tested more than once, we would not recommend the HeiQ-XS but rather one of the test versions with higher test–retest correlation coefficients. However, we believe that, given the small number of items and compared to other test versions with very few items, the HeiQ-XS can be seen as a relatively useful instrument, particularly as its internal consistency and test–retest correlation are similar to those of short test versions of other matrices test.

The HeiQ-S A, HeiQ-S B, and HeiQ-XS all show satisfactory correlations with other tests of general cognitive ability, such as the Berlin Intelligence Structure Test (BIS-S) and the Intelligence Structure Test (I-S-T 2000R), supporting construct validity of the instruments. Even though correlation coefficients were not high, they were in line with expectations and comparable to the ones of the original HeiQ with these measures as well as to the ones of other figural matrices tests, such as the Hagen Matrices Test (Heydasch 2014) or the 18-item RAPM version of Sefcek et al. (2016). Furthermore, comparatively high correlations with high school GPA were observed, suggesting that criterion-related validity was also given. Correlations with the original 48-item version of the HeiQ are also high. With correlations of *r* = 0.96 for both parallel forms of the HeiQ with the original versions, both the HeiQ-S A and HeiQ-S B do not seem to suffer from any loss of information compared to the longer version. Even though the correlation of the HeiQ-XS with the HeiQ is comparatively smaller, with a correlation of *r* = 0.86, this was expected when generating a short form with only six items. The correlation corrected for attenuation of *r* = 1.00 speaks to the excellent validity of the items chosen for the short version.

An additional benefit of the parallel test versions is that distractors can still be analyzed to attain operation-level scores that might give more insight into participants’ cognitive processes and strategies during test taking. Difficulties of test versions are similar for the HeiQ, the HeiQ-S A, and the HeiQ-S B. However, the HeiQ-XS shows to be more difficult. One reason for these findings could be that the HeiQ-S A and HeiQ-S B were chosen on the basis of equally dividing items of the original version to generate two new test forms and achieve the same difficulty in both resulting versions. Thus, it follows that the difficulty of these forms should also be of a similar magnitude. For the HeiQ-XS, however, those items that discriminated the most were chosen, and difficulty was not taken into account. As the validity and reliability of the test are seen as the most important factors, we believe it is not an issue that the test is more difficult compared to the other versions. Furthermore, participants have more (average) time per item to solve the test as a way to reflect the higher difficulty of the included items. As long as all participants work on the same test, a higher difficulty does not influence the informative content of the test as a whole. The relatively high difficulty compared to other tests also allows for application in a wide variety of contexts, especially in research.

Nevertheless, there are also some limitations with the newly developed short forms of the HeiQ. Some subsamples consist of a smaller sample size, such as *N* = 76 for the I-S-T 2000R. While these results are comparable to effects found in similar studies, we acknowledge that the small sample size may limit the generalizability of these findings. It would be interesting to test the different test versions in larger studies. However, with a second sample of *N* = 216 participants that completed the BIS-S, we believe that there is enough statistical power to support the construct validity of all test versions. As already mentioned, some of the reliability estimates of the HeiQ-XS are lower compared to the other test forms. While we believe that the HeiQ-XS is a good measure to serve as a cognitive ability indicator when taken together with a battery of other tests, we would not recommend it in a longitudinal design. This is (a) due to the fact that the retest reliability is, while still good, lower compared to the other test forms and (b) that there will, most likely, be memory effects when only six items are presented. We retested a sample of participants who completed all 48 items of the original versions and found some evidence for retest effects. These retest effects might be even more pronounced when only six items are presented.

To conclude, on top of the possibility of analyzing distractors for their underlying operations and attaining a more detailed sum score, the main goal of the construction of the original HeiQ was to offer a free-to-use, easy-to-implement figural matrices test where distractors could not be eliminated based on superficial features. We also wanted to extend that approach to offer different versions of short forms. In summary, all shorter test versions can be seen as useful and relatively reliable, stable, and valid measures of cognitive ability.

## Figures and Tables

**Table 1:** Demographic of subsamples and assessed measures.

Subsample	*N*	Age		Gender (Female (%))	Population	Assessment Mode	Cognitive Measures	Academic
		*M*	*SD*					
1	155	23.81	5.49	112 (72.3)	University	Online		
2	107	24.18	3.42	72 (67.3)	University of Applied Sciences	Online		GPA
3	126	24.66	4.47	92 (73.0)	University of Applied Sciences	Online		GPA
4	216	26.02	11.18	136 (63.0)	University and general population	In person	BIS-S	GPA
5	79	33.81	13.54	40 (50.6)	General population	Online	I-S-T 2000R RAPM	GPA

Note: GPA: Grade point average for high school and university degree (if applicable). BIS-S = Berlin Intelligence Structure Test Short Form. I-S-T 2000R = Intelligence Structure Test 2000R. RAPM = Raven Advanced Progressive Matrices.

**Table 2:** Overview of the characteristics of the HeiQ, HeiQ-S A and B, and HeiQ-XS.

Test Version	Number of Items	Duration	Balanced Operation Design ^1^
HeiQ (Full Version)	48	60 min	Yes
HeiQ-S A	20	25 min	Yes
HeiQ-S B	20	25 min	Yes
HeiQ-XS	6	10 min	No

^1^ A balanced operation design specifies that all operations are included equally often across the complete item set.

**Table 3:** Test performance of the HeiQ, HeiQ-S A and B, and HeiQ-XS.

Test Version	*M*	*SD*	Range	Skewness	Curtosis	% Solved
HeiQ	26.87	10.68	3–47	0.04	−1.03	55.98
HeiQ-S A	11.74	4.83	1–20	−0.07	−1.02	58.70
HeiQ-S B	11.52	4.64	1–20	0.02	−0.90	57.60
HeiQ-XS	2.54	2.14	0–6	0.36	−1.29	42.33

**Table 4:** Reliability estimates for all test versions.

Test Version	Cronbach’s Alpha (Pairwise)	Split-Half-Correlation	Test-Retest Correlation
HeiQ	0.93	0.88	0.87
HeiQ-S A	0.86	0.76	0.81
HeiQ-S B	0.85	0.75	0.80
HeiQ-XS	0.82	0.70	0.73

Note: Sample size for Cronbach’s Alpha and Split-Half Reliability is *N* = 683. *N* = 205 participants performed the HeiQ twice.

**Table 5:** Correlations between the HeiQ versions, GPA, cognitive ability measures, and the original HeiQ.

Test Version	Highschool GPA	BIS-S	I-S-T 2000R	HeiQ
HeiQ	−0.38 (−0.48)	0.59	0.73	
HeiQ-S A	−0.38 (−0.49)	0.53	0.69	0.96
HeiQ-S B	−0.36 (−0.47)	0.57	0.71	0.96
HeiQ-XS	−0.34 (−0.40)	0.56	0.58	0.87

Note: A lower GPA score indicates a better grade. Numbers in parentheses refer to the correlation of GPA and participants under the age of 24. Sample sizes areas follows: for GPA *N* = 472 (*N* = 264); for BIS-S *N* = 215; and for I-S-T 2000R *N* = 76.

**Table 6:** Model fit indices of the HeiQ, HeiQ-S A and B, and HeiQ-XS.

Test Version	χ2	df	*p*	CFI	RMSEA	SRMR	χ2/df Ratio
HeiQ	3542.68	1127	<.001	0.875	0.056	0.136	3.14
HeiQ-S A	676.29	189	<.001	0.917	0.061	0.116	3.58
HeiQ-S B	771.90	189	<.001	0.892	0.067	0.126	4.08
HeiQ-XS	9.14	14	.822	1.000	0.000	0.023	0.65

Note: CFI = comparative fit index; RMSEA = root mean square error of approximation; SRMR = standardized root nean squared residual; *N* = 683.

## Data Availability

The original data presented in the study are openly available at: https://osf.io/exdfu/?view_only=f9d840e850b141a580c52c5a4121c237 (accessed on 5 June 2024).
